# The Rate-Size Trade-Off Structures Intraspecific Variation in *Daphnia ambigua* Life History Parameters

**DOI:** 10.1371/journal.pone.0081024

**Published:** 2013-12-03

**Authors:** John P. DeLong, Torrance C. Hanley

**Affiliations:** Yale University, Department of Ecology and Evolutionary Biology, New Haven, Connecticut, United States of America; University of Arizona, United States of America

## Abstract

The identification of trade-offs is necessary for understanding the evolution and maintenance of diversity. Here we employ the supply-demand (SD) body size optimization model to predict a trade-off between asymptotic body size and growth rate. We use the SD model to quantitatively predict the slope of the relationship between asymptotic body size and growth rate under high and low food regimes and then test the predictions against observations for *Daphnia ambigua*. Close quantitative agreement between observed and predicted slopes at both food levels lends support to the model and confirms that a ‘rate-size’ trade-off structures life history variation in this population. In contrast to classic life history expectations, growth and reproduction were positively correlated after controlling for the rate-size trade-off. We included 12 *Daphnia* clones in our study, but clone identity explained only some of the variation in life history traits. We also tested the hypothesis that growth rate would be positively related to intergenic spacer length (i.e. the growth rate hypothesis) across clones, but we found that clones with intermediate intergenic spacer lengths had larger asymptotic sizes and slower growth rates. Our results strongly support a resource-based optimization of body size following the SD model. Furthermore, because some resource allocation decisions necessarily precede others, understanding interdependent life history traits may require a more nested approach.

## Introduction

The study of trade-offs underlies much of evolutionary ecology [1]–[4]. Trade-offs arise under constraints because not all competing ends can be maximized simultaneously, and investment of resources in one trait or activity therefore comes at the expense of investment in another. Constraints may arise at multiple levels, such as the constraint of total biomass production imposing a trade-off in the allocation of new biomass to growth versus reproduction, and the constraint of total reproductive effort imposing a trade-off in the size and number of offspring [5]. Trade-offs may equalize fitness across individuals, genotypes, or species, helping to explain the maintenance of standing levels of diversity [6].

Traits that are related by a trade-off typically are negatively correlated. Such negative relationships do not, however, automatically confirm the existence of a trade-off (see discussion in [7]). For example, a negative correlation between growth and reproduction in oaks was caused not by a trade-off in biomass production but by climatic factors influencing both variables simultaneously [8]. To establish the existence of a trade-off, then, it is necessary to identify the constraint and to understand how organisms may have to ‘choose’ between competing ends that each influence fitness.

A major constraint facing any organism is that of environmental resource supply. A supply constraint may produce a trade-off because a given total resource supply can be used by an individual that is either i) large in size with a low mass-specific rate of resource demand or ii) small in size with a high mass-specific rate of resource demand. Such a ‘rate-size’ trade-off would enable the use of all available resources by individuals that show variation in size and mass-specific resource demand. This equalization of resource use could help maintain intraspecific variation in life history traits in a population because all individuals would be exhibiting similar overall resource use even though they vary in size. Here we utilize a simple model that predicts the rate-size trade-off and also allows independent, *a priori* prediction of the slope of the relationship between body size and mass-specific resource demand. Below we describe the model and then discuss how we tested the model using laboratory experiments with *Daphnia ambigua*.

### The supply-demand (SD) model

**Figure 1:**
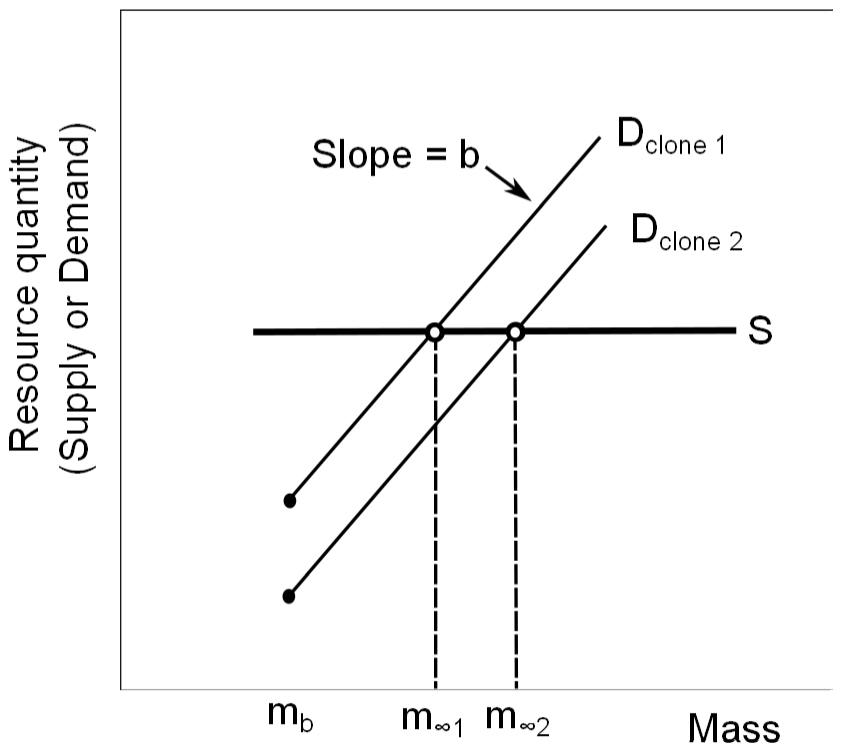
Graphical representation of the supply-demand model. Organisms grow from their natal size, *m*_b_, to their asymptotic or adult size, *m*_∞_, along the demand (*D*) curve. The optimal size is where supply (*S*) equals demand (see text). To maximize resource use, organisms may trade off asymptotic size with mass-specific demand. *Daphnia* may vary in mass-specific demand due to genetic factors such as intergenic spacer (IGS) length, generating changes in asymptotic size because of the supply constraint.

The SD model [9] proposes that the optimal adult body size is that which matches the bodily demand for resources, *D*, with the per capita supply of resources, *S*. The logic of the optimality is that too large a body carries costs because physiological demands go unmet, and too small a body is competitively inferior because it does not maximize resource use. In short, organisms should maximize their use of resources given constraints on resource availability [10], [11]. In the notation of the SD model, organisms begin life at size *m*_b_ and are predicted to reach their asymptotic size *m*_∞_ (or adult size for determinate growers) when supply equals demand (Fig. 1). The demand curve is written as a power function 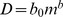, where *b*_0_ reflects mass-specific demand and *b* is a scaling exponent that reflects the change in resource use with size within a species. The supply curve *S* represents the per capita supply rate (which is not necessarily the absolute quantity, but the portion that organisms can actually access) and may take on a variety of shapes and levels in nature. We use a horizontal *S* curve here because we provided all individuals with the same constant food supply in our experiments (see below), but we emphasize that the model can also be developed using non-horizontal *S* curves. In the SD model, the optimal size occurs when *S* = *D*, so we can write 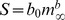 and solve for *m*_∞_, giving 
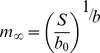
. This simple equation shows that per capita resource supply imposes a trade-off between asymptotic size *m*_∞_ and mass-specific demand *b*_0_; holding *S* constant, an increase in *b*_0_ results in a decrease in *m*_∞_, and vice versa. Taking the log of both sides, we get 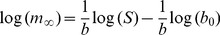
(1).

Equation 1 predicts a negative linear relationship between log(*b*_0_) and log(*m*_∞_) with a slope of 1/*b* with *S* held constant. This prediction was recently tested in an experimental study on the effects of temperature on production rate and cell size in the protist *Actinosphaerium* sp., with a precise match between the predicted and observed slopes of the relationship between mass-specific production rate and cell size [9]. The model also predicts that increasing supply *S* will raise the intercept of the relationship.

Mass-specific resource demand should largely reflect the demands of growth, especially for young organisms that are not yet reproducing, although other activities also contribute to total demand. Higher growth rates cannot be achieved without acquiring more resources, and indeed production is tightly linked to metabolic rate [12], so growth rate must be proportional to *b*_0_. Thus, an equivalent trade-off is that between growth rate and asymptotic size. A growth rate-body size trade-off was previously assumed by Charnov [3] in his development of theory to explain the Beverton-Holt life history invariants [13], yet the constraint governing the trade-off was not identified. Jensen [14] attempted to explain a negative relationship between growth rate and asymptotic size using a simple bioenergetic model of growth. Although the work developed a prediction for the relationship between size and growth rate in fish, the conclusions suffered from three problems. First, the model did not fully isolate body mass (see his Equation 5). Second, the model depended upon several specific assumptions about the scaling of production and maintenance, and therefore is not generalizable to other taxa. And finally, the paper concluded that the constraint governing the trade-off was the necessity of allocating resources to maintenance in the adult stage, even though this would not actually require decreased allocation to growth when young. In contrast to these previous works, the SD model i) identifies the constraint (environmental supply of accessible resources) that governs the trade-off between growth rate and asymptotic size, ii) makes no taxon specific assumptions, and iii) produces an independent, quantitative prediction of the slope between growth rate and asymptotic size.

### Testing the SD model

To test the SD model, we need estimates of demand (growth rate) and asymptotic size. Both of these parameters can be estimated from time series data on growing organisms using growth models such as the von Bertalanffy model [15]: 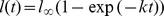, where *l* is length (mm), *l*_∞_ is asymptotic length, and *k* is the growth constant (day^−1^). The growth constant *k* represents mass-specific demand because it is a rate of resource allocation to growth. A negative relationship between the von Bertalanffy growth constant and asymptotic size has been seen widely in fish [3], [13], [14], [16], [17] and salamander larva [18], but it does not always occur (see [19] for an example with eels). The slopes of these published relationships, however, cannot be used to make a robust test of the rate-size trade-off as predicted by the SD model since it is unlikely that resources were held constant across the populations.

Thus, an experimental test with a controlled resource supply and a known source of variation in demand is needed to evaluate the predictions of the SD model. In this study, we conducted such a test by assessing intraspecific variation in life history traits across nearly 150 individuals from 12 *D. ambigua* clones drawn from a single source population and grown under controlled laboratory conditions. *Daphnia*, a model organism for the study of trade-offs, is an important intermediary consumer that displays intraspecific variation in many life history traits including asymptotic size and growth rate [20], [21]. Specifically, we tested the hypothesis that growth rate and asymptotic size would be negatively related, with a slope of -1/*b* across individuals.

The rate-size trade-off may be linked to other trade-offs that involve growth rate, so we also looked at the relationship between growth rate and fecundity. Given a constraint on total biomass production, increased allocation to somatic growth should come at the cost of decreased allocation to reproduction [2], [22]. However, because resource flow through the body begins with the acquisition of food from the environment, it is likely necessary to account for the rate-size trade-off before evaluating the growth-reproduction trade-off. We therefore tested for a trade-off between growth rate and fecundity, and examined the relationship both before and after accounting for variation in growth rate that is related to body size.

Finally, standing variation in life history traits may have a genetic underpinning, so we examined whether variation in mass-specific demand for resources may be linked to genotypic variability in the ribosomal intergenic spacer (IGS) region. The IGS region is important in protein synthesis, and therefore growth and development, because IGS contains regulatory elements central to rRNA transcription and ribosome production [23], [24]. Specifically, the IGS includes a series of tandem subrepeats, each containing putative promoters, enhancers, and terminators that play a critical role in the control of rRNA transcription [25]. IGS sequence length heterogeneity reflects variation in the number of internal subrepeats, and, consequently, in the number of regulatory elements present in the IGS [26]. Therefore, growth rate should be positively correlated with IGS length (the growth rate hypothesis [27]; this correlation has been seen in *Daphnia* [28] and *Drosophila* [29]). Because variation in growth rate, fecundity, and body size may be genetically determined [20], it is possible that the rate-size trade-off could be structured in part by variation in IGS length across genotypes. Therefore, we also tested the growth rate hypothesis by assessing the influence of IGS length on both growth rate and body size.

## Materials and Methods

### Study organisms

Twelve *D. ambigua* clones were established by isolating and hatching ephippia (sexually-produced, diapausing eggs) from the sediment of Bride Lake (East Lyme, Connecticut) [30]. Permission to access Bride Lake was granted by the Connecticut Department of Corrections. To eliminate possible maternal effects, daphniids were reared in the laboratory for 6 months prior to experiments. During this time, all individuals were reared in COMBO medium [31], fed non-limiting quantities (>1 mg C/L) of the high quality food *Scenedesmus obliquus*, and densities were kept relatively constant across clones by putting the same number of adults and juveniles into each culture for weekly changes. The cultures were changed on the same dates and reared in a growth chamber with constant temperature (20°C) and light availability (light:dark cycle 14:10 h).

### Life history measurements

Asymptotic size and growth rate of 10–14 individuals from each of the twelve clones were measured. Animals used in the experiments were third clutch or later neonates, chosen to minimize inter-clutch differences. Each individual was isolated less than 24 h after birth, photographed using Leica IM (version 1.20) for subsequent length and area measurements using Image J [32], and placed in 100 ml COMBO media containing either 1 mg C/L or 0.1 mg C/L *S. obliquus* (high and low food quantity treatments, respectively). A total of 72 and 76 individuals were reared in low and high food conditions, respectively. For the duration of the experiment, food and media were changed daily to maintain constant conditions (constant *S* through time and across clones). Individuals were monitored from birth to fourth clutch under controlled conditions (14:10 h light:dark cycle, 20°C). Daphniids were photographed and subsequently measured five times beginning on day 3 through production of fourth clutch. Day 1 (age<24 h) was excluded to avoid the confounding effects of maternal investment in reproduction. Clutch size was measured by counting the number of eggs or neonates in the brood chamber of the first four clutches, which represents ∼90% or more of the total fecundity of these clones under these conditions [33].

### Intergenic-spacer analsyis (IGS)

To compare IGS length of *Daphnia* clones, 4-8 individuals were isolated from batch cultures. DNA was extracted from each animal using 50 µl 5% Chelex 100 (Bio-Rad); samples were incubated at 55°C for 4–8 h, gently mixed, heated to 98°C for 10 min, vortexed, centrifuged at 14,000 rpm for 2 min, and stored at −20°C. The IGS region of *D. ambigua* was amplified using primers located in the conserved flanking regions – 28S and 18S ribosomal RNA (28S-228F 5′-TGACTCGTTTCGACGTTTTATC and 18S-6223R 5′-AGGATGGAGTGGATCTAAGGAA) (Hanley unpublished data). Each 25 µl reaction contained 2 µl DNA, 0.5 µl of each primer (10 µM), 4 µl of 5× Phusion® GC buffer (7.5 mM MgCl_2_), 1.6 µl of dNTPs (2.5 mM each), 0.6 µl of DMSO, and 0.1 µl of Phusion® DNA polymerase (New England BioLabs). After an initial denaturation step at 98°C for 30 sec, samples were processed through 35 cycles consisting of 98°C for 10 sec, 58°C for 30 sec, and 72°C for 3 min 30 sec, and a final elongation step at 72°C for 10 min. Each sample was subsequently run on 0.75% agarose gel for at least 2.5 h at 80 V to maximize separation between bands. There are many copies of the IGS region of different lengths in any given individual [34]. Therefore, a weighted average of IGS length for each individual was calculated by determining the relative intensity of each band using ImageJ [32]. The mean IGS length for each individual was then used to determine the average IGS length of each clone.

### Data analysis

We fit the von Bertalanffy growth model to each individual growth series using non-linear ordinary least squares regression in Matlab © 2009b. The fitting procedure gave estimates of *k* and *l*_∞_, which was converted to asymptotic mass, *m*_∞_, using the length-to-mass equation for *D. ambigua*: 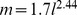, where *m* = mass (µg) [35]. The growth curve for each individual indicates that rearing *D. ambigua* through release of their fourth clutch allowed sufficient time for them to reach their asymptotic size. The relationship between *k* and *m*_∞_ was analyzed using reduced major axis (RMA) regression on log-transformed data following Equation 1 [36]. We also used RMA to analyze the relationship between fecundity and *k*. To control for the relationship between *k* and *m*_∞_, we further analyzed the relationship between the *x*-axis residuals of the *k* versus fecundity relationship (the horizontal deviations from the regression fit). Finally, we analyzed the relationship between mean IGS length and both *k* and *m*_∞_ using general linear models with linear and quadratic IGS terms.

### Predictions

**Figure 2:**
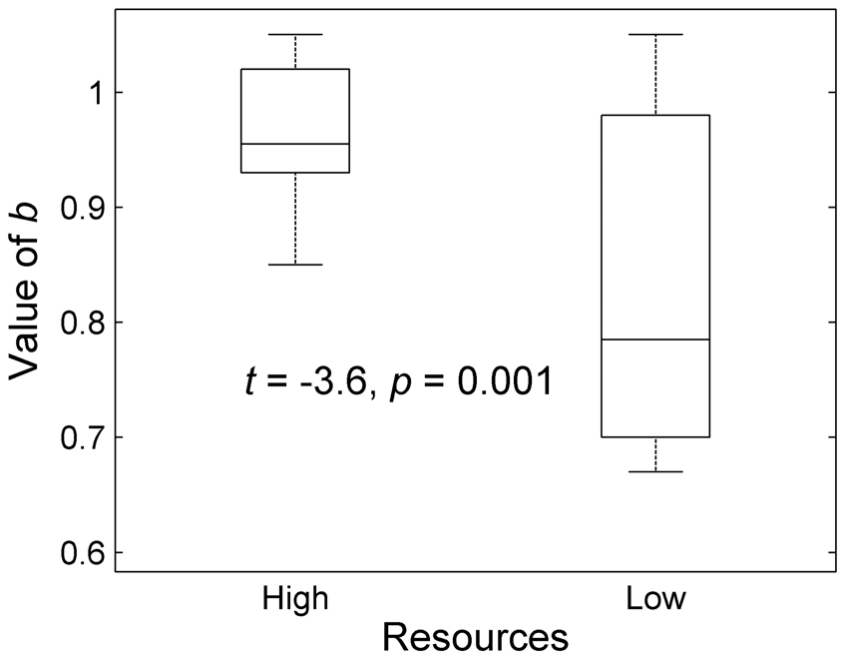
Box plot of the values of the metabolic scaling slope *b* from studies that provided high and low levels of resources. Boxes show median and 25^th^ and 75^th^ percentiles. See Table S1 for details for each study [38], [50]–[58].

The metabolic scaling slope *b* varies from approximately 0.7 to near 1 for various *Daphnia* sp. (Table S1). A notable pattern in this variation is that when resource availability is low (e.g. low food, low phosphorus content, or low oxygen availability), the scaling slope is lower, whereas in nearly all situations where resources are abundant, the slope is closer to 1 (Fig. 2). Resource levels (‘high’ and ‘low’, see Table S1 for details) explain some of the variation in *b* values reported for *Daphnia* (*t* = −3.6, *p* = 0.001). This pattern also occurs for other organisms such as phytoplankton [37] and appears to be related to how resource restrictions of any kind more strongly affect large individuals than small individuals (e.g. [38]). Here we utilize this variation to broaden our test of the SD model predictions. Using Equation 1, we predicted that the slopes of the relationship between *m*_∞_ and *k* in high resource conditions would be −1/0.96≈−1.04 and in low resource conditions would be −1/0.83≈−1.21, based on grouped mean RMA values of *b*. We also conducted an equivalent analysis using a value of *b* pooled across all studies (pooled mean  = 0.91), which predicts a slope of −1.1, and tested this against data for *m*_∞_ and *k* pooled across high and low food levels (Fig. S1). In addition, the difference in supply levels allows us to test the prediction that the intercept of the rate-size trade-off slope would be higher in the high food treatment than in the low food treatment.

## Results

**Figure 3:**
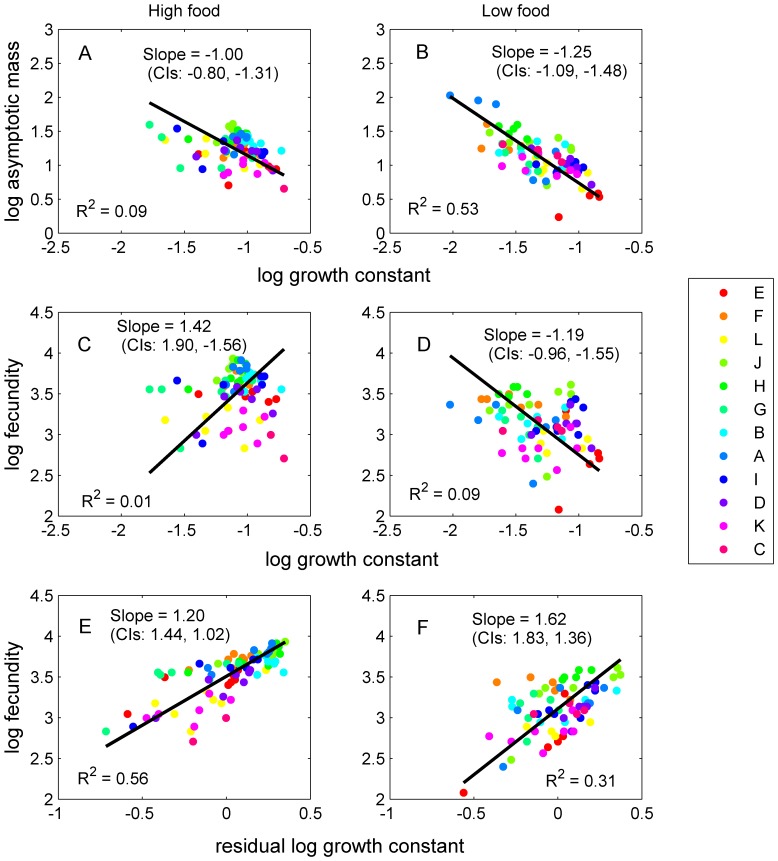
The relationship between asymptotic mass, *m*_∞_, and growth constant, *k*, matches predictions of the supply-demand model under both high (A) and low (B) resource levels. Black lines show RMA regression fits. Fecundity was not related to asymptotic mass under high (C) resource levels but was negatively related to asymptotic mass under low (D) resource conditions. After controlling for the rate-size trade-off, however, using the residual growth rates from A and B, fecundity was positively correlated with the growth constant under both high (E) and low (F) resource levels. *Daphnia* clones are ordered from shortest (E) to longest (C) intergenic spacer (IGS) length.

Our empirical results were in strong agreement with model predictions. Time-series data on body size fit the von Bertalanffy growth model very well, with *R* ^2^ values of the fits from 0.61 to 1.00 (Fig. S1). Across individuals, *m*_∞_ and *k* were significantly negatively correlated (*p*<0.05). Importantly, the slopes were approximately −1/*b* for both high and low resource levels (high resources: predicted slope  = −1.04, observed slope  = −1.00, 95% confidence intervals [CIs]: −0.80 to −1.31; low resources: predicted slope  = −1.21, observed slope  = −1.25, CIs: −1.09 to −1.48). For pooled data, the observed slope (−1.07) was very close to the predicted slope (−1.1; Fig. S2). The intercept of the relationship was higher in the high food treatment than in the low food treatment (high food  = 0.14, CIs: −0.18 to 0.38; low food  = −0.51, CIs: −0.88 to −0.29). For high and low resource levels, asymptotic mass *m*_∞_ and the growth constant *k* varied among individuals and clones (ANOVA; low resource levels: *m*_∞_, *F*_11,60_ = 5.2, *p*<0.001; *k*, *F*_11,60_ = 5.2, *p*<0.001; high resource levels: *m*_∞_, *F*_11,64_ = 7.1, *p*<0.001; *k*, *F*_11,64_ = 2.96, *p* = 0.003; Fig. 3A,B).

There was no evidence for a relationship between log fecundity and log *k* for high resource levels (slope = 1.42, CIs: −1.90 to 1.56; *p*>0.05), but the relationship was negative for low resource levels (slope = −1.19, CIs: −0.96 to −1.55; Fig. 3C,D; *p*<0.05). However, the relationship between *k* and fecundity may have been obscured by the relationship between *k* and *m*_∞_. We accounted for the rate-size trade-off by analyzing *x*-axis residual variation in *k*, and found a strong positive relationship between residual *k* and fecundity (high resource slope = 1.2, CIs: 1.02 to 1.44, *p*<0.05; low resource slope = 1.62, CIs: 1.36 to 1.83, *p*<0.05; Fig. 3E,F). This same pattern held for pooled data (Fig. S2) and so is not simply a result of the high/low resource dichotomy. Thus, some of the variation in growth rate is linked to body size, and some of the remaining variation is linked to fecundity. These results indicate that growth rates vary at multiple levels, first as a trade-off against size, and secondly in parallel to the allocation to reproduction. Although the 95% CIs between low and high resource conditions overlap, there is some suggestion of a relatively higher allocation to fecundity in the low resource than in the high resource conditions due to the steeper slope of the fit in low resource conditions.

**Figure 4:**
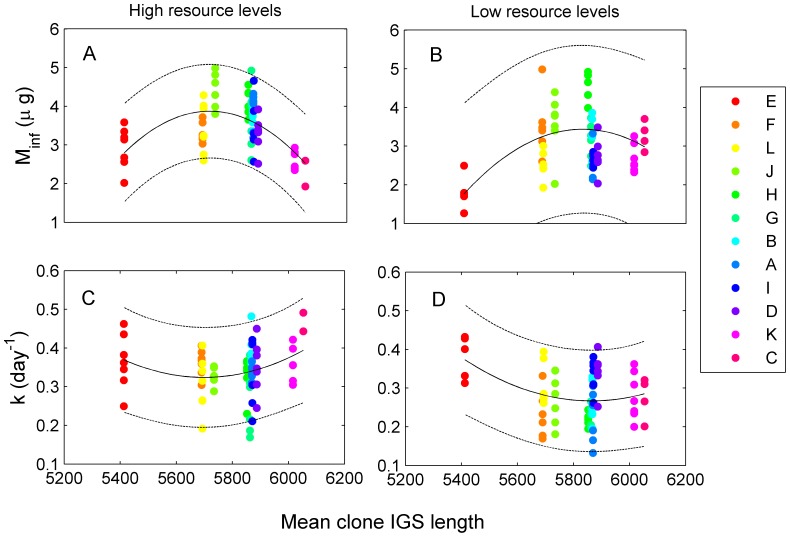
Intergenic spacer (IGS) length was non-linearly related to both asymptotic mass, *m*_∞_, and growth constant, *k*. Intermediate levels of IGS corresponded to relatively high mass and low growth. *Daphnia* clones are ordered from shortest (E) to longest (C) IGS length.

Mean IGS length was nonlinearly related to *m*_∞_ and *k*, with the highest levels of *m*_∞_ and lowest levels of *k* at intermediate values (Fig. 4A–D). Using models with only a linear term, IGS was unrelated to either *m*_∞_ or *k* at high resource levels (*m*_∞_, *F*_1,74_ = 0.12, *p* = 0.73; *k*, *F*_1,74_ = 0.63, *p* = 0.43), but was negatively related to *k* (*F*_1,70_ = 5.83, *p* = 0.02) and marginally positively related to *m*_∞_ (*F*_1,70_ = 3.25, *p* = 0.08) at low resource levels. Using models with a quadratic term, IGS length was significantly related to *m*_∞_ and *k* at both resource levels (high resource levels: *m*_∞_, *F*_2,73_ = 9.62, *p*<0.001; *k*, *F*_2,73_ = 5.92, *p* = 0.004; low resource levels: *m*_∞_, *F*_2,69_ = 5.60, *p* = 0.006; *k*, *F*_2,69_ = 5.66, *p* = 0.005).

## Discussion

Trade-offs arise under constraints that preclude maximal allocation of resources to multiple traits or activities at the same time. Here, we identified a rate-size trade-off in a population of *D. ambigua* under experimentally controlled conditions. Such a trade-off is predicted by the supply-demand (SD) model based on the principle that the optimal body size is that which matches total bodily demand for resources with resource supply (Fig. 1). The SD model predicts that the mass-specific resource demand (in this case considered to be proportional to the growth constant *k*) should be related to asymptotic size *m*_∞_ on logged axes with a slope that is the inverse of the metabolic scaling slope *b*, if the supply curve is horizontal (Fig. 1, Equation 1). This condition was met by providing each individual with the same absolute quantity of food, although it is possible that individuals varied in their ability to access this food. The predicted slope was observed under both high and low resource conditions, with the expected shift in slopes predicted by the change in metabolic scaling slopes between high and low resource conditions (Figs. 2,3 A,B). In addition, as predicted by Equation 1, an increase in supplied food levels was accompanied by a significant increase in the intercept of the trade-off curve. Experimental conditions controlled the *S* curve across clones and time, making these results a robust test of the model predictions. The SD model thus provides an advance over previous theory [3], [14] in its ability to explain standing variation in and correlations among life history traits within populations.

Negative correlations between *k* and *m*_∞_ have been observed previously [3], [14], [16]–[18], but such correlations do not necessarily demonstrate a trade-off [7], [8]. Furthermore, the relationship is not always negative, as seen in a study of *D. middendorffiana* where *k* and *m*_∞_ positively covaried given variation in predation risk and resource levels [39]. By identifying the supply constraint at the individual level, here we were able to show that the observed negative correlation between *k* and *m*_∞_ across individuals does indeed represent a trade-off. In this case, the trade-off occurs within a single panmictic population, indicating that the rate-size trade-off helps to structure standing variation in life history traits. Previously, negative relationships between growth rate and size have been seen primarily across populations. Given that the correlation between *k* and *m*_∞_ is broadly observed, however, the rate-size trade-off may help to explain variation in body size and life history that exist within and among populations. Unexplained variance in the relationship could reflect other important traits, such as potential variation in size-based foraging efficiency across clones [40].

In this experiment, variation in *k* was not driven by environmental factors because all individuals experienced the same temperature and predation regime (no predation risk). Rather, covariation in *m*_∞_ and *k* were linked to variation in IGS length associated with clone identity, as well as other unknown factors. This pattern is predicted by the growth rate hypothesis given the link between IGS length and protein synthesis [29], [41], [42]. However, rather than a sustained positive effect of IGS length on growth rate as expected from previous work, we found a nonlinear effect, with lowest *k* and highest *m*_∞_ at intermediate IGS lengths (Fig. 4). This pattern suggests a potentially complex association between IGS length and the factors driving plasticity in life history traits. Nonetheless, variation in IGS helped to position clones and individuals along the rate-size trade-off curve, where intermediate IGS lengths tended toward the upper left part of the trade-off curve, and long and short IGS lengths occupied the lower right part of the trade-off curve (Fig. 3).

The rate-size trade-off maintains total resource use at optimal levels given the environmental supply, but growth rate may still vary with other traits. For example, growth rate is often linked via trade-offs to reproduction under a total production constraint [12]. In our data, the relationship between *k* and fecundity varied with resource level, being non-significant at high resource levels and negative at low resource levels. This suggests that a growth rate-fecundity trade-off exists when food is scarce (Fig. 3D). Given that some of the variation in *k* is due to the rate-size trade-off and therefore varies to accommodate total resource use, it was necessary to control for the rate-size trade-off to fully understand the link between *k* and fecundity. After accounting for the rate-size trade-off by analyzing the residuals of *k*, we found that the relationship between *k* and fecundity was strongly positive under both high and low resource conditions. Thus, there is no evidence here of a trade-off between growth and fecundity across individuals. However, we expect a growth rate-fecundity trade-off only under a production constraint, so it may be that total production varies across individuals inversely with maintenance, obscuring the trade-off. This pattern could not be seen without first controlling for the rate-size trade-off, which therefore suggests that asymptotic size and growth rate are adjusted to keep resource use constant, and then growth rate continues to vary in response to other demands. For example, it is possible that additional variation in growth rate could be linked to a trade-off with swimming performance, as has been seen in the Atlantic silverside fish *Menidia menidia* [43].

The rate-size trade-off may have a role in maintaining intraspecific variation by making fitness similar across phenotypes, even though variation in size also may influence fitness via other means [44]. Nonetheless, intraspecific variation in size and growth rate due to genetic differences may have important ecological consequences for this population [45]. Ecological effects of body size are myriad but often depend on the allometric expectation that larger individuals use more resources than smaller individuals. This is not the case for the within-population variation in size here, because the rate-size trade-off indicates that growth rate is traded for size to keep resource use levels roughly constant across individuals. Nevertheless, variation in filter size may determine whether clones interact differently with their resource species [46], altering within-population dynamics. Similarly, body size is linked to predation risk [47], potentially creating a complex interaction between how size and growth rate alters the ecological interactions of this population.

The SD model also makes quantitative predictions about the relationship between asymptotic size and resource supply (the slope should be 1/*b*). A positive relationship between food levels and body size has been observed for daphniids [39], as well as other small aquatic invertebrates such as water mites [48]. Such correlations could be compared to predictions from the SD model to determine whether the body size optimization process underlying the rate-size trade-off also explains other patterns of intraspecific size and life history variation, but this has not been done. Recently, however, the SD model has been used to account for variation in body size of the protist *Didinium nasutum* through time by linking it to dynamic changes in the per capita availability of prey [49], suggesting that variation in resource supply in time and space may help to explain broader-scale patterns in body size.

In conclusion, our results indicate that i) the negative relationship between growth rate and asymptotic size in *D. ambigua* represents a trade-off under a supply constraint that precisely conforms to the predictions of the SD model, ii) the covariation between growth rate and asymptotic size in *D. ambigua* has a partly genetic basis in IGS length, and iii) accounting for the rate-size trade-off was necessary for understanding patterns of allocation between growth and reproduction. Finally, we suggest that the rate-size trade-off may play a role in maintaining intraspecific variation by neutralizing resource-based fitness differences, but further work investigating this possibility is needed.

## Supporting Information

Figure S1 **Growth curves for individual daphniids used in this study.** There were 72 individuals in the low food treatment and 76 individuals in the high food treatment. The asymptotic lengths were closely related to the maximum observed lengths (right panel). The black line is the 1:1 line.
(TIF)

Figure S2 **Analysis of life history traits of 12** ***Daphnia ambigua*** **clones using data pooled across high and low food levels.** **A**. Using all data on the metabolic scaling exponent *b* for *Daphnia* species, the predicted slope for the relationship between log(*k*) and log(*m*_∞_) is −1.1, and the observed slope is −1.07. **B**. Fecundity is unrelated to the growth constant *k*. **C**. After controlling for the rate-size trade-off in **A**, however, a positive relationship between fecundity and residual *k* emerges. These results for the pooled data mirror the separate results for high and low food conditions reported separately in the main text.
(TIF)

Table S1Ordinary least squares (OLS) and reduced major axis (RMA) regressions for the power law scaling of metabolic rate with body size for various *Daphnia* sp. Data are the exponent, *b*, from the standard metabolic scaling model 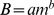, where *B* =  metabolic rate, *a* is a pre-factor, and *m* is body mass. Units of *B*, *a*, and *m* vary across studies, but *b* is comparable across studies. Data are shown with 95% confidence intervals where available. RMA regressions were conducted on digitized data from original source or converted to RMA equivalent by dividing the OLS *b* by the reported correlation coefficient *r*.(DOC)
