# Supplementary figures and images for: The Rate-Size Trade-Off Structures Intraspecific Variation in Daphnia ambigua Life History Parameters
Source: PLoS One. 2013 Dec 3;8(12):e81024. doi: 10.1371/journal.pone.0081024 (PMC3849075; doi:10.1371/journal.pone.0081024)

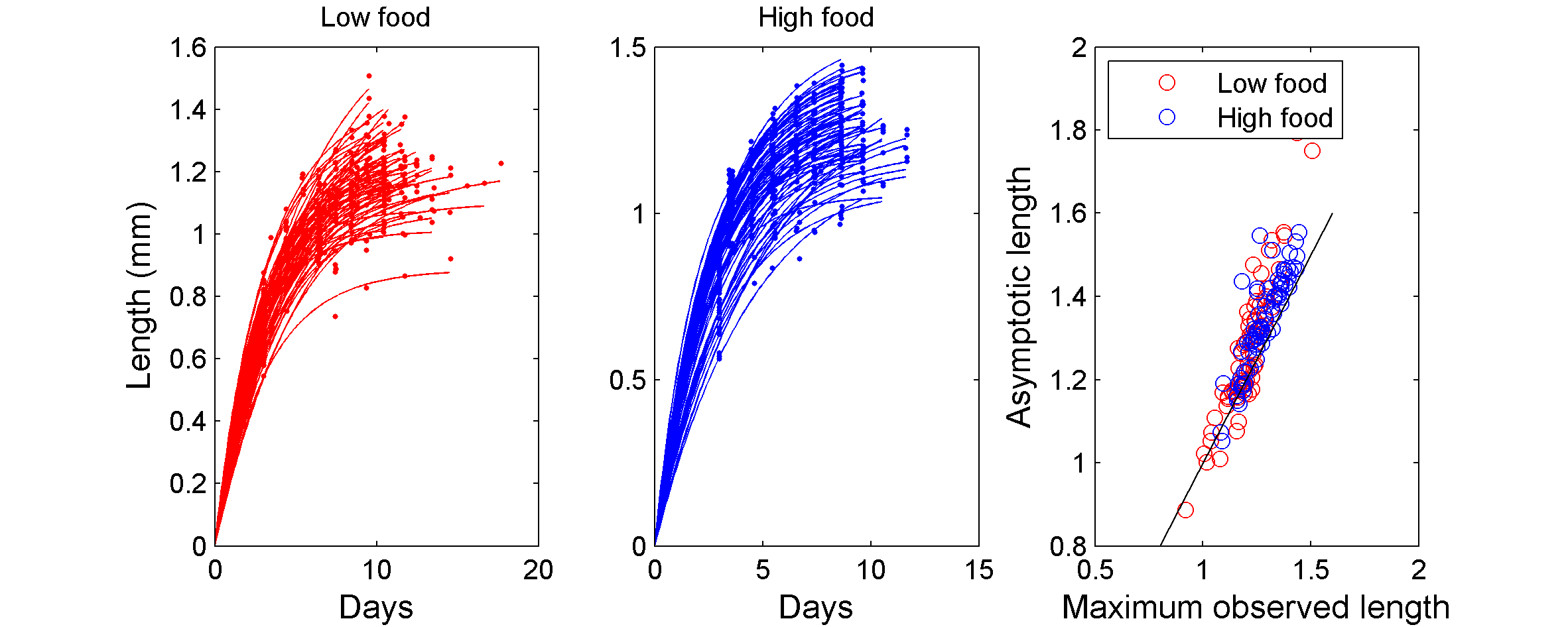

Supplement: Figure S1 — Growth curves for individual daphniids used in this study. There were 72 individuals in the low food treatment and 76 individuals in the high food treatment. The asymptotic lengths were closely related to the maximum observed lengths (right panel). The black line is the 1:1 line. (TIF) [file pone.0081024.s001.tif]

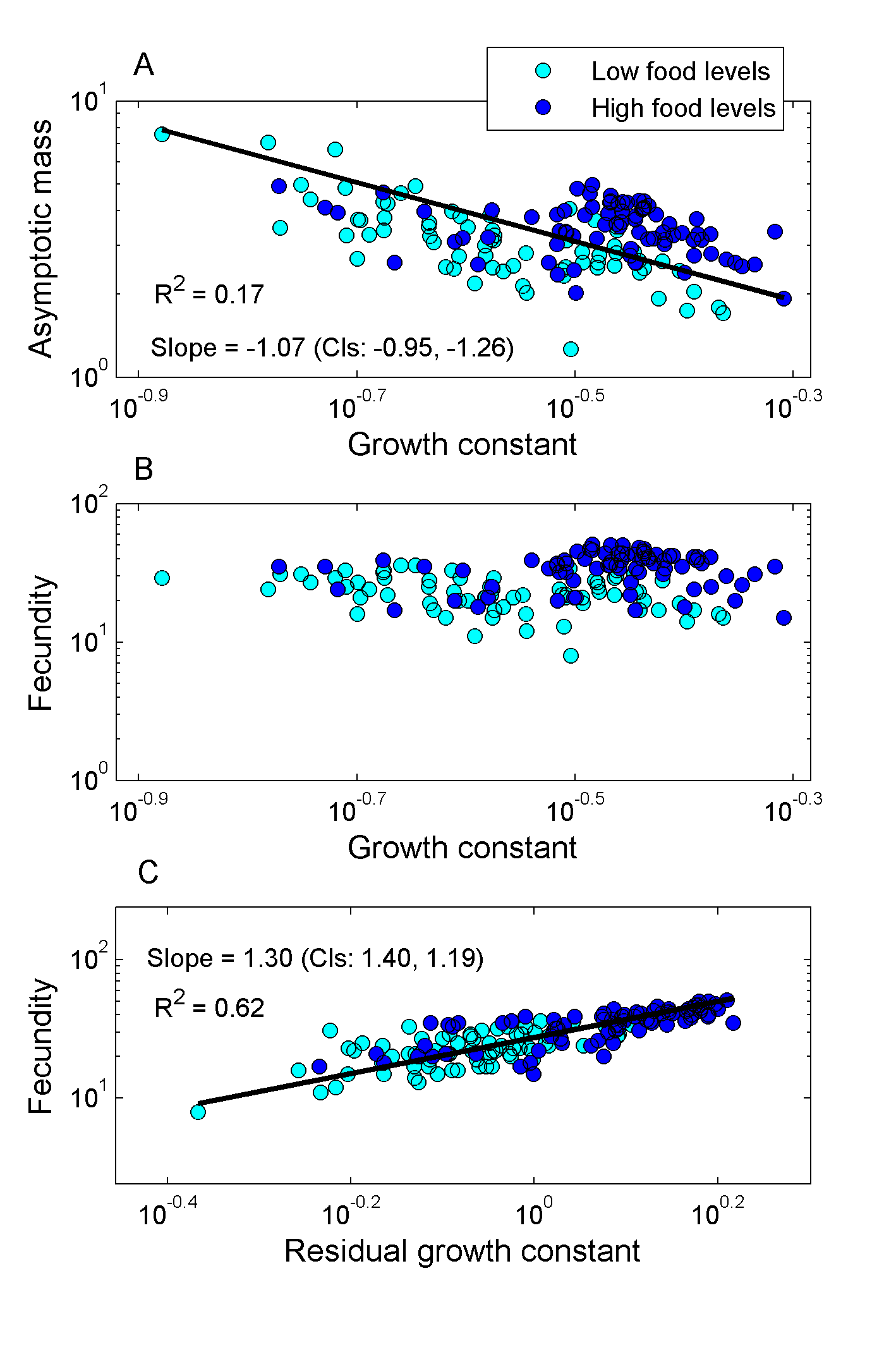

Supplement: Figure S2 — Analysis of life history traits of 12 Daphnia ambigua clones using data pooled across high and low food levels. A. Using all data on the metabolic scaling exponent b for Daphnia species, the predicted slope for the relationship between log(k) and log(m ∞) is −1.1, and the observed slope is −1.07. B. Fecundity is unrelated to the growth constant k. C. After controlling for the rate-size trade-off in A, however, a positive relationship between fecundity and residual k emerges. These results for the pooled data mirror the separate results for high and low food conditions reported separately in the main text. (TIF) [file pone.0081024.s002.tif]
