# Supplementary material for: The Rate-Size Trade-Off Structures Intraspecific Variation in Daphnia ambigua Life History Parameters
Source: PLoS One. 2013 Dec 3;8(12):e81024. doi: 10.1371/journal.pone.0081024 (PMC3849075; doi:10.1371/journal.pone.0081024)
Supplement: Table S1 — Ordinary least squares (OLS) and reduced major axis (RMA) regressions for the power law scaling of metabolic rate with body size for various Daphnia sp. Data are the exponent, b, from the standard metabolic scaling model , where B = metabolic rate, a is a pre-factor, and m is body mass. Units of B, a, and m vary across studies, but b is comparable across studies. Data are shown with 95% confidence intervals where available. RMA regressions were conducted on digitized data from original source or converted to RMA equivalent by dividing the OLS b by the reported correlation coefficient r. (DOC) [file pone.0081024.s003.doc]

**Table S1**. Regressions for the power law scaling of metabolic rate with body size for various *Daphnia* sp.

| **Species** | **Source** | **Level** | ***b*_OLS_** | ***b*_RMA_** | R^2^ |  |
| --- | --- | --- | --- | --- | --- | --- |
| **Food quantity** |  |  |  |  |  | Notes |
| *D. pulex* | Richman (1958) | High | 1.03 (0.9 to 1.11) | 1.05 (0.98 to 1.18) | 0.96 | Fed *ad libitum* and then held 24 h before measurement. Original data were reanalyzed, excluding three individuals with large broods, with length converted to dry weight using the formula of (Lynch et al. 1986) |
| *D. magna* | Schindler (1968) | High | 0.923 (0.90 to 0.95) | 0.93 (0.91 to 0.95) | 0.99 | Held 12 hours without food, indicated small broods, original data reanalyzed |
| *D. pulex* | Buikema Jr. (1972) | Unknown | 0.86 (SE: 0.12) |  |  | From rearing light levels; ‘unacclimated’ animals; food levels unspecified but animals taken from stock tank without being provided food *ad libitum*; no R2 reported and therefore no corresponding RMA value available; masses generated from linear conversion of length to weight which biases *b* values low |
| *D. pulex* | Buikema Jr. 1972 | Unknown | 0.64 (SE: 0.07) |  |  | From rearing light levels; ‘acclimated’ animals kept under light conditions at least two molt cycles; food levels unspecified; no R2 reported and therefore no corresponding RMA value available; masses generated from linear conversion of length to weight which biases *b* values low |
| *D. magna* | Kersting & Leeuw-Leegwater (1976) | Low | 0.78 (0.62 to 0.95) | 0.85 (0.77 to 0.97) | 0.84 | Animals fed only every three days, acclimated 15 minutes without food before measurements |
| *D. ambigua* | Armitage & Lei (1979) | Low | 0.58 (-) | 0.67 |  | Field conditions without *ad libitum* food supply |
| *D. pulex* | Lynch et al. (1986) | High | 1.09 (-) | 1.10 | 0.99 | Oxygen consumption measured with food present with control bottles to correct; respiration corrected for broods |
| *D. ambigua* | Lynch et al. (1986) | High | 0.90 (-) | 0.95 | 0.89 | Oxygen consumption measured with food present with control bottles to correct; respiration corrected for broods |
| *D. magna* | Glazier (1991) | High | 0.95  (0.92 to 0.98) | 0.96 | 0.98 | Well fed and fasted 24 h prior to measurement; respiration corrected for broods |
| *D. magna* | Glazier & Calow 1992 | High | 0.95 (-) | 0.97 | 0.98 | Clone S-1; high-food rations were 1.5 mg C L^-1^; respiration corrected for broods |
| *D. magna* | Glazier & Calow 1992 | High | 0.92 (-) | 0.94 | 0.98 | Clone F; high-food rations were 1.5 mg C L^-1^; respiration corrected for broods |
| *D. magna* | Glazier & Calow 1992 | Low | 1.00 (-) | 1.05 | 0.95 | Clone S-1; low-food rations were 0.3 mg C L^-1^; respiration corrected for broods |
| *D. magna* | Glazier & Calow 1992 | Low | 0.94 (-) | 0.98 | 0.96 | Clone F; low-food rations were 0.3 mg C L^-1^; respiration corrected for broods |
| **Food quality (P content)** |  |  |  |  |  |  |
| *D. magna, D. lumholtzi, D. pulex*, and *D. obtusa* | Jeyasingh (2007) | High | 0.83  (0.81 to 0.85) | 0.90 | 0.85 | Cross species analysis; P-enriched food |
| *D. magna, D. lumholtzi, D. pulex*, and *D. obtusa* | Jeyasingh (2007) | Low | 0.67  (0.66 to 0.69) | 0.72 | 0.86 | Cross species analysis; P-depressed food |
| *D. magna* | McFeeters & Frost (2011) | High | 1.01 (-) | 1.02 | 0.99 | P-rich conditons; food given in non-limiting quantities (2-6 mg C L^-1^) |
| *D. pulex* | McFeeters & Frost (2011) | High | 1.02 (-) | 1.03 | 0.99 | P-rich conditons; food given in non-limiting quantities (2–6 mg C L^-1^) |
| **Oxygen levels** |  |  |  |  |  |  |
| *D. magna* | Kobayashi 1982 | Low | 0.68 (0.62 to 0.74) | 0.70 (0.64 to 0.75) | 0.96 | High hemoglobin levels evidence of low oxygen levels, original data reanalyzed |
